# Supplementary material for: The interactome of intact mitochondria by cross-linking mass spectrometry provides evidence for coexisting respiratory supercomplexes
Source: Mol Cell Proteomics. 2017 Dec 8;17(2):216–32. doi: 10.1074/mcp.RA117.000470 (PMC5795388; doi:10.1074/mcp.RA117.000470)
Supplement: Supplemental Data [file supp_RA117.000470_133922_0_supp_23978_fzffwf.zip › spectra_annotation/mito_spectra_annotation/86-1-4-1-19-1.pdf]

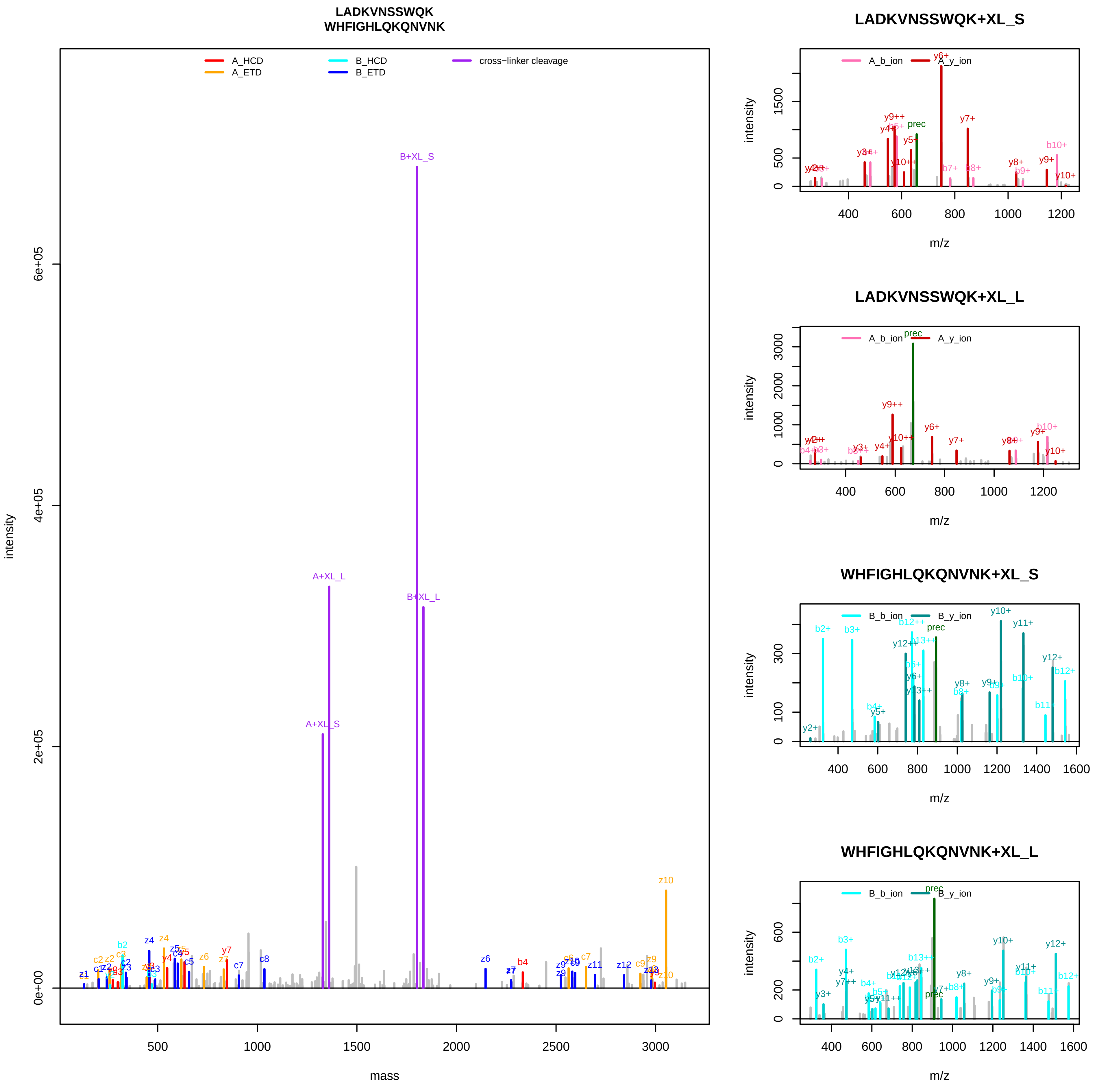

LADKVNSSWQK
WHFIGHLQKQNVNK
A_HCD
A_ETD
B_HCD
B_ETD
cross−linker cleavage
B+XL_S
A+XL_L
B+XL_L
A+XL_S
z10
intensity
mass
0e+00
2e+05
4e+05
6e+05
500
1000
1500
2000
2500
3000
LADKVNSSWQK+XL_S
A_b_ion
A_y_ion
y6+
y7+
prec
b10+
y9++
intensity
m/z
LADKVNSSWQK+XL_L
A_b_ion
A_y_ion
prec
y9++
y6+
y7+
y10+
intensity
m/z
WHFIGHLQKQNVNK+XL_S
B_b_ion
B_y_ion
y10+
y11+
b2+
b3+
b12++
prec
y12+
b12+
intensity
m/z
WHFIGHLQKQNVNK+XL_L
B_b_ion
B_y_ion
prec
b3+
b2+
y10+
y12+
intensity
m/z
